# Mycobacterial and HIV Infections Up-Regulated Human Zinc Finger Protein 134, a Novel Positive Regulator of HIV-1 LTR Activity and Viral Propagation

**DOI:** 10.1371/journal.pone.0104908

**Published:** 2014-08-21

**Authors:** Ronald Benjamin, Atoshi Banerjee, Kannan Balakrishnan, Ramya Sivangala, Sumanlatha Gaddam, Sharmistha Banerjee

**Affiliations:** 1 Department of Biochemistry, School of Life Sciences, University of Hyderabad, Hyderabad, Telangana, India; 2 Immunology Department, Bhagwan Mahavir Medical Research Centre, A.C. Guards, Hyderabad, Telangana, India; 3 Department of Genetics, Osmania University, Hyderabad, Telangana, India; Institut de Pharmacologie et de Biologie Structurale, France

## Abstract

**Background:**

Concurrent occurrence of HIV and Tuberculosis (TB) infections influence the cellular environment of the host for synergistic existence. An elementary approach to understand such coalition at the molecular level is to understand the interactions of the host and the viral factors that subsequently effect viral replication. Long terminal repeats (LTR) of HIV genome serve as a template for binding trans-acting viral and cellular factors that regulate its transcriptional activity, thereby, deciding the fate of HIV pathogenesis, making it an ideal system to explore the interplay between HIV and the host.

**Methodology/Principal Findings:**

In this study, using biotinylated full length HIV-1 LTR sequence as bait followed by MALDI analyses, we identified and further characterized human-Zinc-finger-protein-134 (hZNF-134) as a novel positive regulator of HIV-1 that promoted LTR-driven transcription and viral production. Over-expression of hZNF-134 promoted LTR driven luciferase activity and viral transcripts, resulting in increased virus production while siRNA mediated knockdown reduced both the viral transcripts and the viral titers, establishing hZNF-134 as a positive effector of HIV-1. HIV, *Mycobacteria* and HIV-TB co-infections increased hZNF-134 expressions in PBMCs, the impact being highest by mycobacteria. Corroborating these observations, primary TB patients (n = 22) recorded extraordinarily high transcript levels of hZNF-134 as compared to healthy controls (n = 16).

**Conclusions/Significance:**

With these observations, it was concluded that hZNF-134, which promoted HIV-1 LTR activity acted as a positive regulator of HIV propagation in human host. High titers of hZNF-134 transcripts in TB patients suggest that up-regulation of such positive effectors of HIV-1 upon mycobacterial infection can be yet another mechanism by which mycobacteria assists HIV-1 propagation during HIV-TB co-infections. hZNF-134, an uncharacterized host protein, thus assumes a novel regulatory role during HIV-host interactions. Our study provides new insights into the emerging role of zinc finger proteins in HIV-1 pathogenesis.

## Introduction

Human immunodeficiency virus-1 (HIV-1) infection progressively compromises the immune system against opportunistic infections and malignancies [1], [2], leading to acquired immune deficiency syndrome (AIDS). HIV epidemic is further complicated by syndemic interactions with other opportunistic infections, especially tuberculosis (TB) [3], [4]. Studies from our lab and others clearly indicate that HIV patients with TB co-infection have higher viral titers than HIV patients without TB co-infection [4], [5]. The molecular mechanisms behind these coalitions are largely unknown. A fundamental question pertaining to the pathogenesis of HIV, both under single and co-infection is to identify and understand the interactions of the host and the viral factors that subsequently affect the virus replication [6], [7], [8], [9].

Laboratory observations, supported by clinical sampling of co-infected tissues have led to tractable hypotheses explaining some of the basis for exacerbation of HIV infection by *Mycobacteria* [5]. Higher HIV replication in *M.tb* stimulated macrophages [10], [11] suggested that HIV replication gets augmented at the sites of *M.tb* infection, leading to increased pathology. The monocytes infected with *M.tb* are known to release TNF-α which induces NF-κB mediated activation of long terminal repeats (LTR) of HIV thus promoting viral gene transcription [12], [13]. Additionally, *M.tb* has been shown to abolish the inhibitory impact of host factors like, C/EBP-β, on HIV propagation thus explaining the synergism [14].

HIV-1 Long Terminal Repeats (LTR), that flank the functional genes in the viral genome [15] is largely responsible for regulating early events in HIV-1 lifecycle, rendering it an ideal system to explore the interplay between HIV-1 and the host. It resembles a typical eukaryotic promoter that functions as a template to bind trans-acting regulatory cellular and viral proteins [16], [17]. Apart from the viral regulatory proteins, Tat, Nef and Vpr, LTR is also regulated by host proteins, like Sp1 and GATA that are constitutively expressed in a broad range of HIV-1 permissive cells [18], [19], [20], [21]. Some regulatory factors, like NF-κB, TNF-α and NF-AT are inducible [22], [23], [24], hence influence HIV-1 LTR under specific environmental stimuli [25], [26]. There are also LTR inhibitory host factors like OTK-18 [27], tumor suppressor p53 [28], lipopolysaccharide-binding protein-1 (LBP-1) [29] and Yin Yang1 (YY1) [30]. An exhaustive list of host factors interacting with HIV-1 LTR is summarized by Pereira *et.al* [16]. These regulatory factors may vary in response to stimuli like hyperthermia, oxidative stress and infections such as that of mycobacteria [23], [31], [32], [33]. There are emerging evidences that HIV-TB coalition may also be explained by *M.tb* factors promoting HIV-1 LTR activity. IG5, a T lymphocyte cell line (Jurkat cell line stably expressing luciferase reporter gene under the control of HIV promoter, when co-cultured with *M.tb* infected monocytes from healthy or HIV-infected patients, showed 5 to 7 folds increase in the LTR transcriptional activity [34]. Several mycobacterial components, indirectly, through activation of host factors, like TNF-α-NF-κB signaling pathway or TNF-α along with IL-10 can influence HIV-1 LTR activity [12], [13], [35].

In this study, we identified human Zinc finger protein 134 (hZNF-134) as one of the LTR binding proteins from astrocyte cell lysates using biotinylated full length LTR as bait (Figure S1, S2 and File S1). According to UniProt database, hZNF-134 is classified as a Kruppel C2H2-type zinc-finger transcriptional regulator (UniProt ID: P52741) with 11 C2H2-type zinc finger domains (Figure S3); however, its precise function in human cells is still not clear. Over-expression of hZNF-134 increased while siRNA mediated knockdown reduced both the viral RNA levels and HIV-1 titers thus validating it as a positive regulator of LTR and HIV-1 production. The impact of HIV-1 and mycobacterial infections on the expression of hZNF-134 was studied in PBMCs and the observations were substantiated by using tuberculosis patient samples. With this study, we propose that up-regulation of hZNF-134 by mycobacteria may be one of the molecular mechanisms explaining the high HIV-1 titers observed during HIV-TB co-infections [4], [36]. We also put forward that the uncharacterized host protein of undeciphered function in the host, assumes a novel regulatory role during host-pathogen interaction.

## Results

### Human Zinc Finger protein 134 (hZNF-134) bind to HIV-1 LTR and localized to the nucleus of the cell

Human Zinc finger protein 134 (hZNF-134) was identified through MALDI analyses as one of the proteins binding to HIV-1 LTR in pull-downs assays from astrocyte cell lysates using biotinylated HIV-1 LTR immobilized onto Streptavidin agarose beads (Figure S1, S2 and File S1). UniProt database classified hZNF-134 as a Kruppel C2H2-type zinc-finger transcriptional regulator (UniProt ID: P52741) with 11 C2H2-type zinc finger domains (Figure S3). Though hZNF-134 was initially identified from the astrocyte cell lysate, the transcript levels of hZNF-134 were present in different HIV-1 permissive cell lines like glioblastoma, T-lymphocytes and monocytes (Figure S4). hZNF-134, therefore, was not a neural cell specific factor.

**Figure 1:**
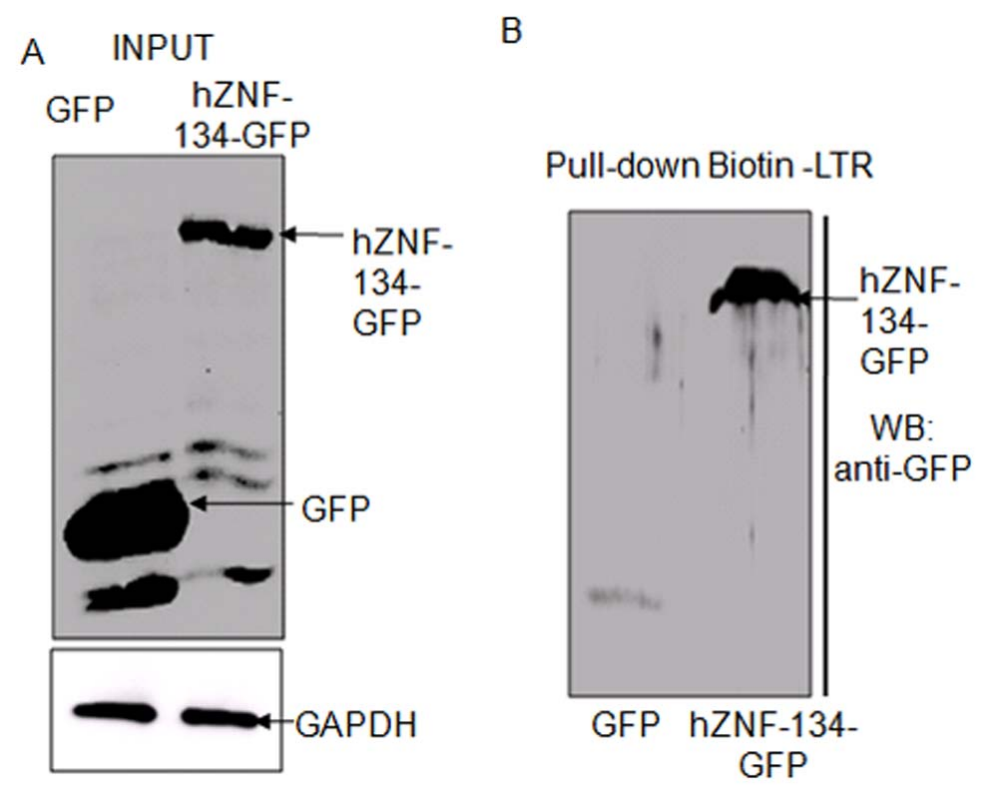
hZNF-134 could bind to HIV-1 LTR. (A) GFP or hZNF-134-GFP fusion proteins were transiently expressed in HEK293T cells. After 48 hours post transfection, cell lysates were prepared and Western blot with anti-GFP antibody was performed to check for the expression of GFP or hZNF-134-GFP. GAPDH was used as a loading control. (B) Transiently expressed GFP or hZNF-134-GFP in HEK293T cells were used for pull-down assays with biotinylated LTR. Western blot analyses of the pull-down samples using anti-GFP antibody showed that biotinylated LTR could capture hZNF-134-GFP successfully but GFP alone was not captured by LTR. Pull-down experiments were performed more than three times.

For further validation of the interaction of hZNF-134 with HIV-1 LTR and its characterization, hZNF-134-GFP fusion protein was over-expressed in HEK293T cells and the expression was checked by Western blot using anti-GFP antibody (Figure 1A). GAPDH was used as a control to ensure that equal amounts of cell lysates were used for the pull-down assays (Figure 1A). The biotinylated HIV-1 LTR was then used to pull-down transiently expressed hZNF-134-GFP from these cells. Despite lower expression of hZNF-134-GFP as compared to GFP alone, hZNF-134-GFP could be specifically pulled down by biotinylated HIV-1 LTR as evident by the presence of hZNF-134 specific band in WB (Figure 1B). These observations confirmed that hZNF-134 could indeed bind with HIV-1 LTR under our conditions.

**Figure 2:**
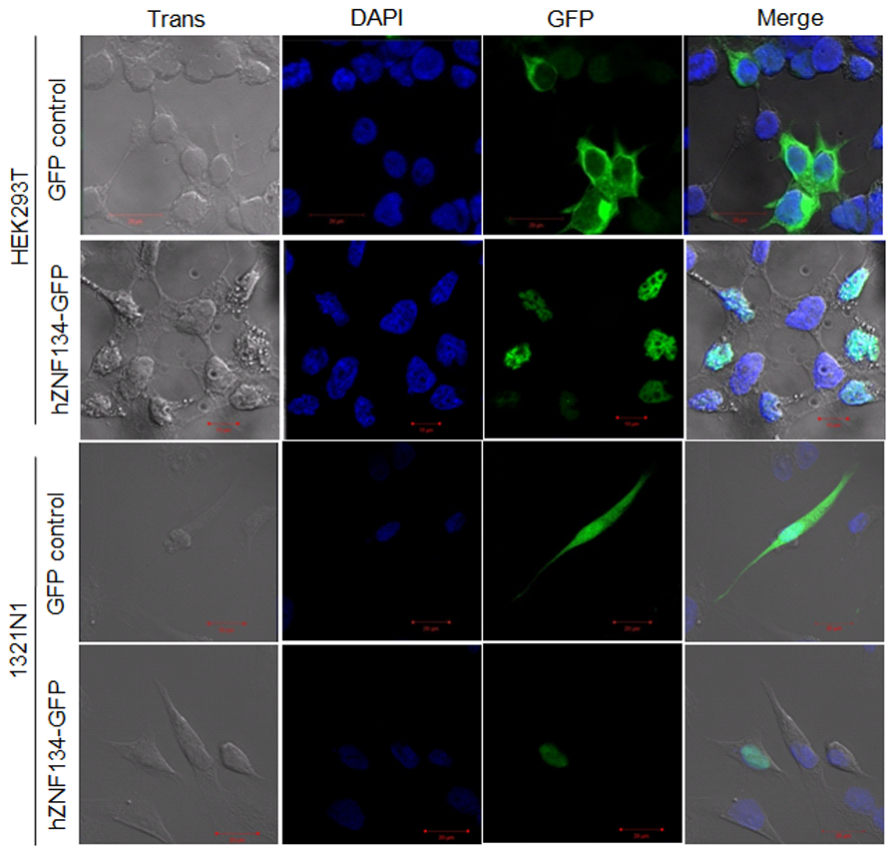
hZNF-134-GFP is localized in the nucleus of HEK293T and Astrocytoma 1321N1 cells. HEK293T and 1321N1 cells were transfected with expression plasmids pEGFP-C3 or ZNF-134-GFP-C3. The cells were fixed after 48 hours and visualized under confocal microscopy. Panels show Trans, DAPI, GFP, and Merged images. hZNF-134-GFP protein was localized in the nucleus of HEK293T and 1321N1 cells whereas GFP control showed both nuclear and cytoplasmic localization. Localization was confirmed by 3 independent experiments.

The sub-cellular localization of hZNF-134 was next determined by confocal microscopy in two cell lines, HEK293T and 1321N1. These cells were transfected for the transient expression of either hZNF-134-GFP fusion protein or GFP alone. hZNF-134-GFP (Figure 2, row 2 and 4, green) co-localized with DAPI-positive nuclei (Figure 2, row 2 and 4, merged) indicating its localization in the nuclei of these cells. The control GFP signal was localized primarily in the cytoplasm of HEK293T cells, but was observed in both the nucleus and the cytoplasm of 1321N1 (Figure 2, row 1 and 3). Therefore, it was inferred that hZNF-134 localized to the nuclei of these cell lines.

### hZNF-134 increased HIV-1 LTR mediated transcription and viral titers

**Figure 3:**
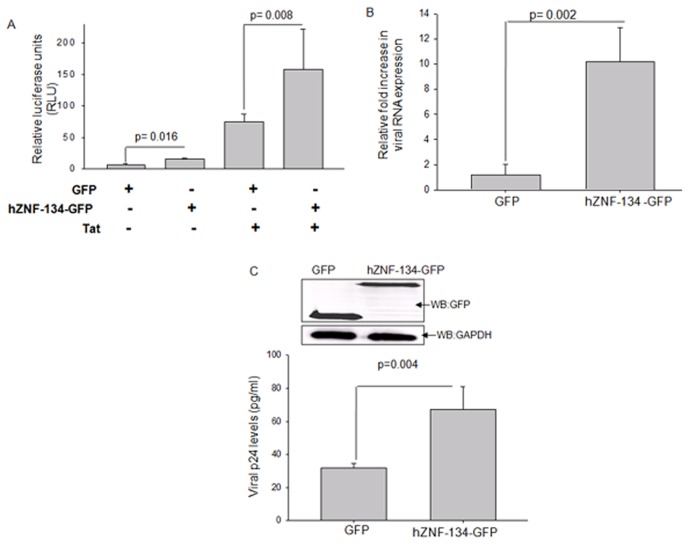
hZNF-134 over expression enhanced the transcriptional potential of LTR and increased viral titers. (A) Luciferase activity was measured in the presence or absence of Tat following transfection with pEGFP-C3 or ZNF-134-GFP-C3 vector into HEK293T cells along with the reporter construct, pLTR-luc. Luciferase activity was measured in terms of relative luminescence units (RLU) from HEK293T cell lysates after 48 hours and plotted as fold increase in the luciferase activity. Cells over-expressing hZNF-134-GFP showed increased luciferase activity when compared to GFP alone. (B) pNL4-3 was transfected in HEK293T cells with either GFP or hZNF-134-GFP. After 48 hours, the viral transcription was measured by qRT-PCR. The plot shows that viral transcripts increased in the presence of over-expressed hZNF-134. (C) HEK293T cells over-expressing either GFP or hZNF-134-GFP were transfected with pNL4-3 vector and after 48 hours scored for viral p24 by ELISA. Inset shows the Western blot confirming the over-expression of either GFP or hZNF-134-GFP in HEK293T cells. For loading control, expression of GAPDH was detected by Western blots in both the cases. Over-expression of hZNF-134 increased viral titers. All the transfection efficiencies were normalized to pRL-TK transfections. All experiments were done more than three times and error bars represents mean ± SD. Student's *t*-test was performed and *p<0.05 was considered significant.

Having confirmed the interaction between hZNF-134 and HIV-1 LTR, we further assessed the impact of this interaction on the transcription strength of HIV-1 LTR by using luciferase based reporter construct with LTR as the promoter (pLTR-luc) [37]. hZNF-134–GFP-C3 or pEGFP-C3 vectors were co-transfected with pLTR-luc construct into HEK293T cells. 48 hours post transfection; cells were lysed and assayed for luciferase activity. Vector pRL-TK was used to normalize the transfection efficiencies. pLTR-luc in the presence of hZNF-134–GFP showed statistically significant (p = 0.016) increase in the luciferase activity by 2 folds when compared to the vector control (Figure 3A). Since the viral protein Tat is one of the major determinants of the LTR activity [38], [39], we also scored the impact of hZNF-134 interaction with HIV-1 LTR in the presence of Tat. It was observed that hZNF-134 in the presence of Tat increased the luciferase activity by 2 folds when compared to Tat co-expressed with GFP alone (Figure 3A). Though Tat alone is capable of increasing the luciferase activity to a magnitude of several folds, we observed that hZNF-134 was further able to potentiate the luciferase activity by 2 folds in the presence of Tat (p = 0.008) (Figure 3A).

The effect of hZNF-134 over-expression was then quantified both at viral transcript levels and at levels of final viral output (Figure 3B and 3C). For this, HEK293T cells were transfected with pro-viral DNA pNL4-3 and either hZNF-134-GFP-C3 or empty pEGFP-C3 vector. Transient expression of hZNF-134-GFP was confirmed by Western blot (Figure 3C inset). A near 10 fold increase in the expression levels of viral transcripts were observed when hZNF-134-GFP was transiently expressed as compared to GFP alone (Figure 3B, p = 0.002). The final viral output in terms of p24 equivalent was measured from culture supernatant after 48 hours of pNL4-3 transfection by p24 ELISA. It was clearly evident that the over-expression of hZNF-134 significantly increased (p = 0.004) the viral production in HEK293T cells by 2 folds (Figure 3C). All studies were normalized with pRL-TK which served as the control for the transfection efficiencies.

Summing together, these experiments indicated that hZNF-134, a host protein, binds to HIV-1 LTR, thereby increasing the LTR-driven transcriptional activity that could be correlated with increase in the viral titers. Therefore, hZNF-134 is a positive effector of HIV-1 production.

### siRNA mediated knockdown of hZNF-134 decreased viral transcript levels and viral titers

**Figure 4:**
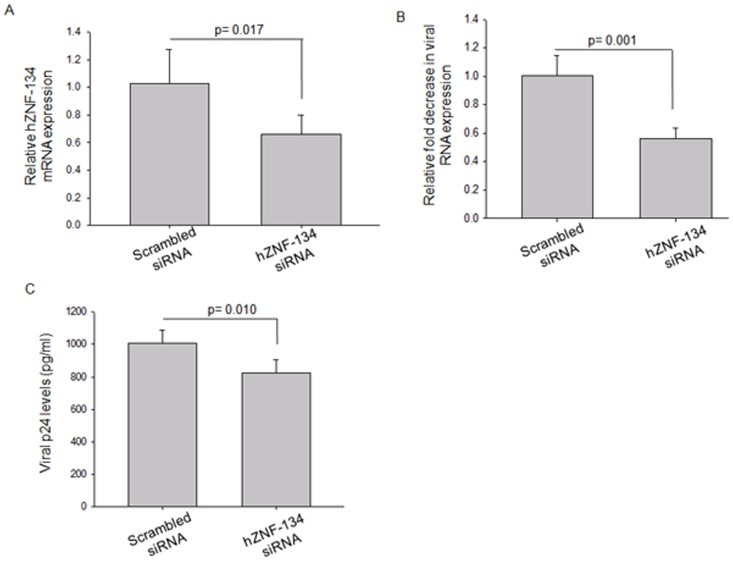
siRNA mediated knockdown of hZNF-134 decreased the viral transcript levels and the final viral titer output measured by p24 ELISA. (A) qRT-PCR showing reduced transcript levels of hZNF-134 after siRNA mediated knockdown. Scrambled siRNA was used as a control. 50picomoles of each siRNA were used. The transcript levels were normalized to the β-actin levels. (B) pNL4-3 was transfected in HEK293T cells treated with 50 picomoles of scrambled siRNA or hZNF-134 siRNA. After 48 hours, the viral transcription was measured by qRT-PCR. The plot shows that viral transcripts decreased following the knockdown of hZNF-134. (C) Viral p24 levels were measured in pNL4-3 transfected HEK293T cells after treatment with 50picomoles of hZNF-134 specific or scrambled siRNA. All experiments were done more than three times and error bars represents mean ± SD. Student's *t*-test was performed and *p<0.05 was considered significant.

The over-expression experiments were further supported by siRNA mediated knockdown studies. Endogenous hZNF-134 was knocked down using specific siRNA, followed by which both the viral transcripts and HIV-1 titers were measured. A scrambled siRNA was used as a control for the experiments. The cells were treated with 50 picomoles of either hZNF-134 specific siRNA or scrambled siRNA and checked for cell viability by MTT assay (Figure S5). Once confirmed that 50 picomoles of siRNA (Set1-sigma Figure S5) against hZNF-134 were not detrimental for HEK293T cells, the siRNAs were validated for their potential to knockdown the levels of endogenous hZNF-134 by qRT-PCR (Figure 4A). A 36±5% knockdown of hZNF-134 at the transcript level was observed as compared to the control experiment (Figure 4A). To confirm that *hznf-134* siRNA was not targeting other genes, we compared the levels of *gapdh* transcript in both the controls and the experimental sets normalized to *β actin*. No significant difference in the *gapdh* gene expression was observed when siRNA against hZNF-134 was used (Figure S6). After 24 hours of siRNA treatment, cells were transfected with proviral DNA pNL4-3. After 48 hours of pNL4-3 transfection, viral transcripts were measured by qRT-PCR and p24 equivalent of viral titers, by p24 ELISA, were quantified. It was observed that 36±5% knockdown of hZNF-134 resulted in 43±10.3% decrease in the viral transcripts (Figure 4B). Viral p24 levels were lowered significantly by 17.5±8% upon partial knockdown of hZNF-134 when compared to scrambled siRNA treated cells (Figure 4C).

With these complimentary experiments involving over-expression and siRNA mediated knockdowns of hZNF-134, we showed that hZNF-134 acted as an activator of LTR-mediated transcription and a positive regulator of viral propagation.

### HIV-1 and mycobacterial infections increased the expression of hZNF-134

**Figure 5:**
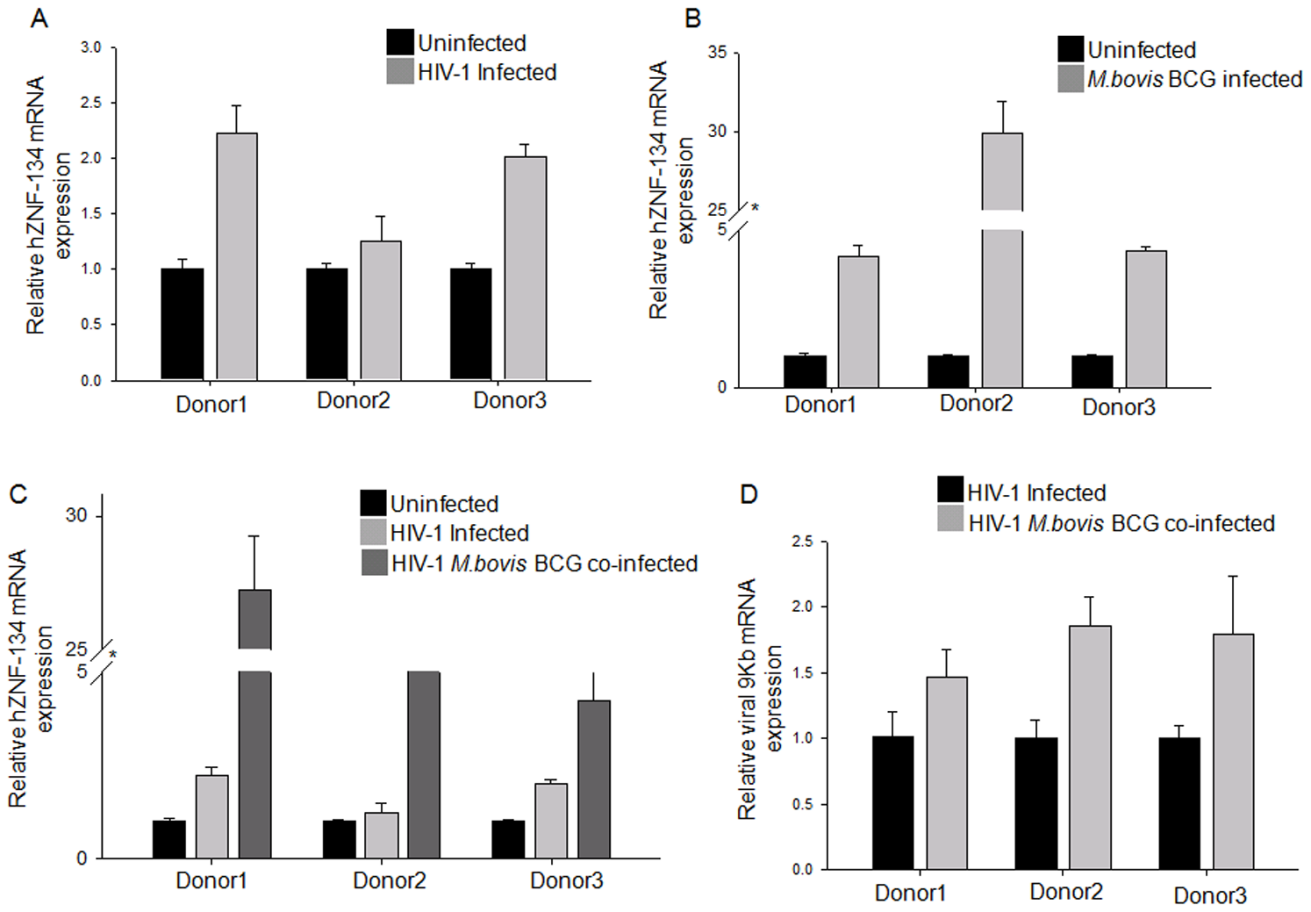
HIV-1 and *Mycobacterium bovis*BCG infections increased the transcript levels of hZNF-134. PBMCs were isolated from three healthy donors and infected either with HIV, *Mycobacterium bovis*BCG or both. The levels of hZNF-134 was measured by qRT-PCR and compared to uninfected controls. (A) hZNF-134 transcript levels upon HIV infection. (B) hZNF-134 transcript levels upon *Mycobacterium bovis*BCG infection. (C) Comparison of hZNF-134 transcript levels upon HIV and HIV-*Mycobacterium bovis*BCG co-infections. Uninfected cells were used as controls. The transcript levels were normalized to transcript levels of β-Actin. (D) The relative expression of 9 kb viral transcript was measured in HIV and HIV-*Mycobacterium bovis*BCG co-infections. The transcript levels were normalized to transcript levels of β-Actin. All experiments were done in triplicate and error bars represent mean ± SD.

We next checked the changes in the endogenous levels of hZNF-134 upon infections through qRT-PCR in PBMCs from healthy donors (n = 3). The PBMCs were isolated and infected with NL4-3 virus (30 ng/ml equivalent of p24). Total RNA was isolated 48 hours post infection for qRT-PCR analysis. HIV-1 infection increased the hZNF-134 levels considerably in all the cases (Figure 5A). The PBMCs were then infected with *M.bovis*BCG (1∶50 MOI) alone or in the presence of NL4-3 representing HIV-TB co-infection. Mycobacterial infection increased hZNF-134 transcript levels at the least 4 folds (Figure 5B), many folds higher than HIV infections. For co-infection, the cells were first infected with NL4-3 virus followed by *M.Bovis*BCG, thus simulating a condition where HIV infected person gets a secondary infection of opportunistic mycobacteria. During NL4-3-*M.bovis*BCG co-infection, the transcript levels of hZNF-134 were observed to be higher than HIV infection alone, suggesting that mycobacterial infection had a predominant impact on upregulation of hZNF-134 (Figure 5C). To correlate the increased levels of hZNF-134 with increase in the LTR activity, thereby, HIV replication, the levels of viral transcripts were scored by qRT-PCR during HIV mono-infection and HIV-*M.bovis*BCG co-infection. We observed that *M.bovis*BCG co-infection in the background of HIV increased the viral transcript levels in all the donors studied in our condition (Figure 5D). These preliminary observations indicated that hZNF-134, which is a positive regulator of LTR driven transcription and HIV-1 propagation, is upregulated during HIV and mycobacterial infections, with mycobacterial infection playing a dominant impact during co-infections.

### Tuberculosis patients registered higher levels of hZNF-134 as compared to healthy controls

**Figure 6:**
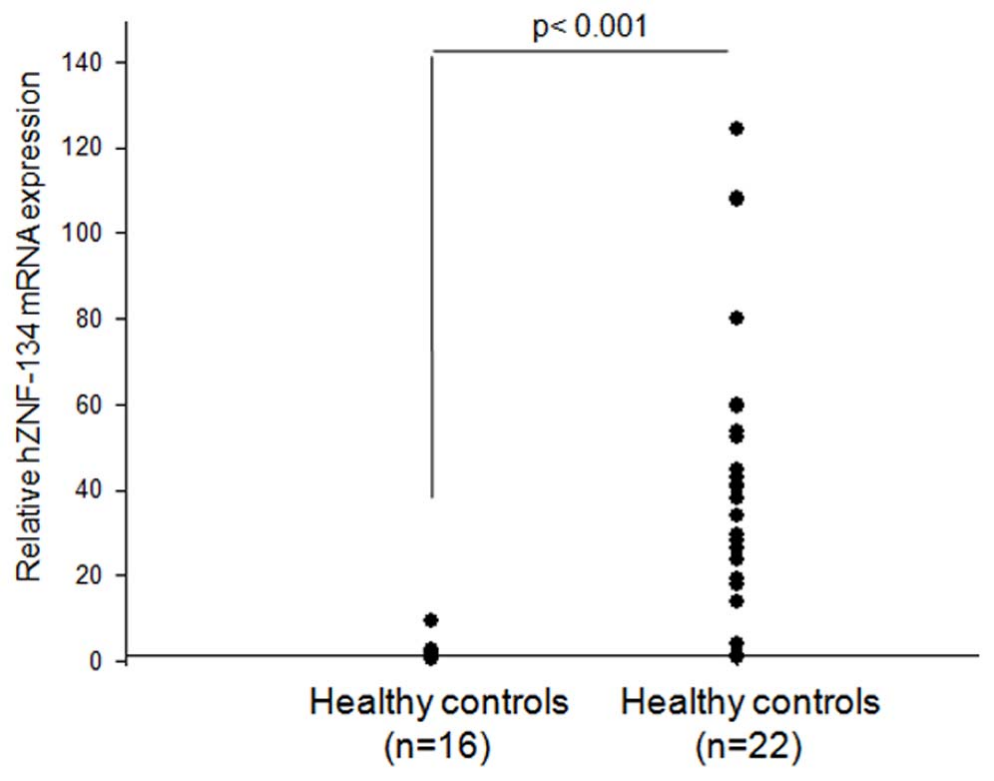
TB patients registered high transcript levels of hZNF-134. PBMCs were isolated from 22 TB patients and 16 healthy controls. RNA was isolated and converted to cDNA followed by qRT-PCR using specific primers for hZNF-134. TB patients registered tremendously high levels of hZNF-134 transcripts when compared to healthy controls. *p<0.05 was considered significant.

The infection assays in PBMCs strongly indicated that between HIV and mycobacteria, mycobacteria had a predominant effect on hZNF-134 expression. To corroborate these results, we checked the transcript levels of hZNF-134 in tuberculosis patients. The TB patients recruited under the study were primary TB cases with no past history of tuberculosis and had not undergone any TB therapy before (referred to as naïve). The PBMCs from these TB patients (n = 22) were analyzed for hZNF-134 RNA levels by qRT-PCR and were compared to that of the healthy controls (n = 16). hZNF-134 was highly up-regulated in all the TB patients when compared to the healthy controls (Figure 6), which strongly supported the infection assays in cultured PBMCs.

Overall, this study established that hZNF-134, a host factor which could bind to HIV-1 LTR, promoting its transcription strength, thereby increasing the viral titers, was upregulated in tuberculosis patients. Increase in the levels of such a positive effector of HIV by *Mycobacterium tuberculosis* may be one of the molecular mechanisms by which mycobacteria elevates viral load in HIV-TB patients. This uncharacterized human host protein, of hitherto unknown function, thus, assumes a regulatory role during host-pathogen interactions.

## Discussion

HIV, like many retroviruses, integrates its genome into the host chromosome and then onwards the expressions of viral proteins are carried out entirely by the host machinery [40], [41], [42]. HIV-1 is regulated by host factors at various stages of its life cycle; one of the initial critical step of which is Long Terminal Repeat (LTR) mediated transcriptional regulation of viral RNA. LTR serves as a template for binding trans-acting viral and cellular factors that either positively or negatively influences its transcriptional activity, thereby, deciding the fate of HIV pathogenesis. In this paper, we report a Zinc finger protein-134 (hZNF-134) that interacts with HIV-1 LTR and promotes its transcriptional activity.

In a quest to identify new LTR interacting partners, we used biotinylated LTR as bait and proceeded to characterize them functionally in the context of HIV pathogenesis. We adopted affinity column based pull-down in which biotinylated LTR was immobilized to the streptavidin beads and incubated with cell lysates to capture DNA interacting host partners. hZNF-134 was identified from the astrocyte cell lysates which was used for the pull-down assays. Though identified from astrocyte cell lysates, we checked the expression of hZNF-134 in different HIV-1 permissive cell lines like glioblastoma, T-lymphocytes and monocytes. We observed that the transcript levels of hZNF-134 were present in all the cell lines (Figure S4). hZNF-134, therefore, cannot be claimed as a neural cell specific factor. Amongst the other proteins that were captured in the pull-downs was Ku80. Ku80 is an established ubiquitously expressed HIV-1 LTR binding protein which has been reported to influence LTR mediated transcriptional activity [43]. There are several reported cellular transcription factors with zinc finger motifs that bind and influence the activity of HIV-1 LTR, such as Sp-1, GATA, YY1, LBP-1 and OTK-18 [18], [19], [27], [29], [30]. Collectively, these studies have established the impact of zinc finger motifs on HIV LTR activity and HIV pathogenesis. Attempts are being made to design specific zinc finger motifs to target the LTRs, for instance, to prevent integration of LTR flanking viral genome [44], [45]. Unveiling more such proteins will add new facets to the molecular understanding of HIV-host interactions. Hence, hZNF-134, a zinc finger protein, was selected for further characterization in this paper.

hZNF-134 belonged to the Kruppel C2H2-type Zinc-finger protein family (Figure S3) that constitutes one of the largest family of transcription regulators. However, the physiological function of hZNF-134 inside a human host cell is yet undefined. The genomic location of hZNF-134 in human is chromosome 19 (Location: 19q13). This is remarkable for the fact that chromosome 19 is also the most frequent region for retroviral integration [42], [46]. Proximity of hZNF-134 gene to the retroviral integration hotspots raises a question if it has any involvement in the integration of viral genome apart from the observed positive regulation of HIV-1 LTR mediated transcriptional activity in our study. The Kruppel associated box (KRAB) domain is absent in hZNF-134. Most commonly, C2H2 proteins with KRAB domain, function as transcriptional repressors [47]. With the absence of this repressor domain, one may expect that hZNF-134 will not exhibit repressor properties. This was evident in our study where hZNF-134 emerged as an activator of HIV-1 LTR mediated transcription.

To check the physiological relevance of the hZNF-134-LTR interaction, we looked for the role of hZNF-134 in increasing the LTR mediated transcription activity. We observed that following the over-expression of hZNF-134, there was an increase in the luciferase activity in terms of relative luminescence unit (Figure 3A). As Tat is the major viral mediator of LTR driven transcription, the LTR-driven luciferase activity of hZNF-134 was also evaluated in the presence of Tat. hZNF-134 together with Tat enhanced the LTR mediated activity by 2 folds (Figure 3A). When the viral transcripts levels from pNL4-3 were quantified in the absence or in the presence of hZNF-134-GFP, a near 10 fold increase was observed (Figure 3C). A 10 fold increase in the viral transcripts upon over-expression of hZNF-134 established it as a strong positive regulator of viral transcription; however, the enormous 10 folds increase in the viral transcript was eventually translated to a 2 fold increase in the final viral titers (Figure 3B–C). This can be explained by the fact that the final viral output is additionally regulated at several post transcriptional levels, like viral RNA transport, splicing, translation, packaging, encapsidation, among others [48], [49].

Over-expression of hZNF-134-GFP increased HIV-1 production, while partially silencing hZNF-134 by specific siRNA decreased the viral transcripts and consequently the p24 equivalent of viral titers (Figure 3 and 4). It was noticed that the majority of the cells died whenever there was an attempt to improve the knockdown of hZNF-134 by increasing the dosage of hZNF-134 specific siRNA (Figure S5). Several alternate approaches and protocols with different transfection reagents were used, but any concentration above 50 picomoles of hZNF-134 specific siRNA led to the loss of cell viability, though the cells treated with scrambled siRNA remained viable (Figure S5). Assuming that the siRNA designed against hZNF-134 may be targeting other essential proteins non-specifically, we also used alternate siRNAs, including the commercially available siRNA (SantaCruz). However, in our conditions, the cells could tolerate only 50 picomoles of hZNF-134 siRNA that led to 36±5% decrease in the transcript levels (Figure 4A). The knockdown of hZNF-134 quantified by qRT-PCR was normalized to the transcript levels of β-actin. GAPDH was used as yet another control to rule out non-specific targeting by hZNF-134 siRNA (Figure S6). As the specific function of hZNF-134 in the host is still not known, it is possible that hZNF-134 may participate in crucial cell functions making it an important factor. It is important to note here that several zinc finger DNA binding proteins participate in critical cellular functions like DNA replication [50], apoptosis [51], inflammation [52] etc that are decisive in cell survival. Studies to decipher the physiological relevance of this apparently essential protein are under progress. With respect to HIV-1 infection, the decrease of 36±5% in transcript levels of hZNF-134 resulted in 17.5±8% decrease in HIV titers, establishing its involvement in increasing HIV viral load.

Once the function of hZNF-134 with respect to HIV was established, we checked the fate of this protein upon infection. Previous studies have shown that HIV LTR activity can be induced by mycobacterial infection, both by its proteins and cell wall components. Mycobacterial protein Rv1168c is known to interact with TLR-2, resulting in the downstream activation of NF-κB signaling pathway, thereby activating the LTR in monocytes/macrophages [13]. Mannose-capped lipoarabinomannan, a cell wall component of *M.tb* is also known to influence the HIV LTR activity by inducing the expression of TNF-α in the monocytoid cells [12]. TNF-α in both soluble and membrane form is sufficient to induce LTR activity, as addition of neutralizing antibody against TNF-α abrogated the LTR activity [53], [54]. TNF-α is well known to mediate NF-κB activation which translocates to the nucleus and binds to the conserved NF-κB binding sites located in the enhancer region of the LTR. TNF-α along with IL-10 has been reported to contribute to the activation of LTR in the cell lines carrying latent HIV infection [35]. One of the other intriguing observations in this study was the up-regulation of hZNF-134 at the transcript levels upon both HIV-1 infection and *M.bovis*BCG infection. PBMCs from healthy donors, when infected with HIV-1 or *M.bovis*BCG or both, showed increased levels of hZNF-134 transcripts as compared to un-infected controls (Figure 5). It could be inferred that the infection increases the expression of hZNF-134 in immune cells, expanding its horizon as a regulator at the host-pathogen interface. This can be of particular significance during mycobacterial co-infection alongside HIV-1 infection since mycobacterial infection is the most common opportunistic infection in HIV-1 patients [5]. Recently published observations from our lab indicated that the viral loads were always higher in HIV patients with TB co-infections [4]. All the donors showed comparatively higher levels of hZNF-134 transcripts during co-infection than HIV mono infection, though the degree varied (Figure 5C). When we compared the induction of hZNF-134 upon mycobacterial and HIV infections separately, it became apparent that Mycobacterial infection increased hZNF-134 transcript levels many folds higher than HIV infections (Figure 5B), suggesting that mycobacterial infection had a predominant impact on upregulation of hZNF-134. We supplemented these observations by measuring the levels of hZNF-134 in primary TB patients with no past history of TB. To our surprise, TB patients (n = 22) showed an average 40 fold increase in the hZNF-134 transcript levels as compared to the healthy controls (Figure 6). A systematic population study involving well-defined cohort of HIV and HIV-TB co-infected patients are under progress. We further intend to extend similar study with respect to other opportunistic infections in HIV patients. This will help us in explaining why HIV disease progression is accelerated in the presence of opportunistic infections. Additionally, we observed that the transcript levels of hZNF-134 are elevated in neural cell lines, Astrocytoma 1321N1 and Glioblastoma GO-G-CCM, after HIV-1 infection (Figure S7). Further investigations may reveal the role of hZNF-134 in HIV associated neuronal disorders. These inputs encourage further studies on the role of hZNF-134 in tuberculous meningitis in both TB and HIV-TB patients. The field based analyses of the levels of hZNF-134 in TB, HIV and HIV-TB co-infected patients may reveal its importance as a putative biological marker. These observations, thus, open up new avenues for deciphering the molecular mechanism behind the coalition between mycobacteria and HIV.

## Materials and Methods

### Ethics Statement

Blood from volunteers were collected at Immunology Department, Bhagwan Mahavir Medical Research Centre, Hyderabad. The tuberculosis patients enrolled at Mahavir Hospital for PPM-DOTS were studied. This study and all the related protocols were approved by Institutional Ethics Committee for Biomedical Research at Bhagwan Mahavir Medical Research Centre under Indian Council MR, Govt. of India IEC guidelines (ECR/450/Inst/AP 2013) and the Institutional Biosafety Committee, UoH (UH/SLS/IBSC/Review/SB-R-11) under Department of Biotechnology, Govt. of India. Informed written consents were taken from the participants before enrolling them for the study.

### Cell lines

The cell lines used for the study: HEK293T (human embryonic kidney cell line) obtained from Dr. Reddy's Institute of Life Sciences, Hyderabad; Astrocyte 1321N1 (human neural cell line) [55] and Glioblastoma GO-G-CCM (human neural cell line) [56] obtained from Prof. Anand K Kondapi, University of Hyderabad were maintained in Dulbecco's Modified Eagle Medium (Gibco, USA). SUP-T1 (human T cell lymphoblastic lymphoma) obtained from Dr. S. Jameel. ICGEB, Delhi and THP-1 (human acute monocytic leukemia cell line) obtained from NCCS, Pune were maintained in Rosewell Park Memorial Institute Medium (RPMI)-1640 (Gibco, USA) [57]. The media were supplemented with 10% fetal bovine serum (Gibco, USA), 100 U of penicillin/ml and 100 µg of streptomycin/ml (HiMedia Laboratories, India) at 37°C with 5% CO_2_. The cells were harvested when 80% confluent and were washed with Phosphate buffered saline (PBS). Cell lysates were prepared in PBS having 0.5% Nonidet P-40 (Sigma Aldrich, USA) and protease cocktail (Sigma Aldrich, USA).

### Plasmids and constructs

*ZNF-134-GFP-C3*: *hznf-134* gene was amplified from cDNA reverse transcribed from Astrocytoma 1321N1 using primers ZNF-GFP-FP and ZNF-GFP-RP and cloned into XhoI and BamHI sites of the vector pEGFP-C3 (Figure S8). The clones were confirmed by sequencing (Eurofins, India). Primer list is given in Table S1. *pLTR-Luc* [37] (a gift from Dr. Debashish Mitra, NCCS, Pune, India); *pNL4-3*: a full length replication and infection competent chimeric DNA (Gene Bank: AF324493) [58] *; pcTat* (a gift from Prof Anand K Kondapi), *pRL-TK* (Promega, USA) and *pEGFP-C3* (Clonetech, USA) were used for the study.

### PBMC isolation and infections with NL4-3 and *Mycobacterium bovis*BCG

All experiments were performed in the facilities approved for Mycobacterial and HIV cultures by University of Hyderabad Institutional Biosafety Committee under Department of Biotechnology, Govt. of India (UH/SLS/IBSC/Review/facilities F-60 & F-70). Blood was collected from healthy donors and TB patients in Heparin vacutainers (Becton Dickinson vacutainer system, USA). Blood was collected from TB patients at the point of detection before the beginning of any TB therapy. PBMCs were isolated using Histopaque (GE Healthcare Life Sciences, USA). Blood was diluted with PBS in the ratio of 1∶1 and was gently layered over the Histopaque. The samples were centrifuged at 800 g for 30 minutes at room temperature. The PBMC layer was separated carefully and transferred to a new tube. The PBMCs were rinsed twice with PBS. The cells were rested in the RPMI-1640 supplemented with 10% FBS and 100 U of penicillin/ml and 100 µg of streptomycin/ml. Prior to any infection, the PBMCs were activated with PHA (10 ug/ml). PBMCs were infected for 4 hours with *Mycobacterium bovis*BCG (MOI = 1∶50) grown in 7H9 media (Himedia Laboratories, India) supplemented with 10% OADC (Himedia Laboratories, India) and 0.05% Tween-80 (Sigma-Aldrich, USA). NL4-3, which is a HIV replication and infection competent chimeric DNA, stocks were prepared as previously described [59]. In brief, pNL4-3 was transfected into HEK293T cells by calcium phosphate method and supernatant was collected and filtered through 0.45 µM filter and precipitated using PEG 10000. The virus was quantified by HIV-1 p24 ELISA according to manufacturer's protocol and 30 ng/ml p24 equivalent of NL4-3 was used to infect cell lines or PBMCs for 2 hours in presence of 8 µg/ml polybrene (Sigma-Aldrich, USA).Viral titers were measured by p24 ELISA (Advanced BioScience Laboratories Inc, USA) as per manufacturer's protocol. In general, an average of ∼15800 HIV equates to 1 pg per p24 gag [60].

### Biotinylated HIV-1 LTR amplification and pull-down assays

The details of pull-downs from astrocytoma 1321N1 cell lysates using biotinylated HIV-1 LTR are given as File S1, Figure S1 and S2. Pull-down assays were also performed using cell lysates of HEK293T cells transiently expressing hZNF-134-GFP or GFP protein. The cell lysates were incubated with the biotinylated LTR immobilized to streptavidin agarose beads. The beads were washed 5 times with 1X Saline-sodium citrate buffer before adding SDS loading dye. The samples were fractionated on 10% SDS-PAGE and transferred onto nitrocellulose membrane (Pall Life sciences, USA). The Western blot was performed using 1∶1000 dilution of mouse anti GFP-antibody (SantaCruz Biotechnology Inc., USA) followed by secondary detection with 1∶2000 dilution of HRP conjugated goat anti-mouse IgG antibody (SantaCruz Biotechnology Inc., USA). This was detected using Pierce ECL western substrate (Thermo Scientific, USA) and visualized using VersaDoc gel imaging system (BioRad, USA).

### Confocal Microscopy

The adherent HEK293T and 1321N1 cells were seeded and transfected with ZNF-134-GFP-C3 or pEGFP-C3 vectors using Lipofectamine LTX and plus reagent (Invitrogen, USA). The cells were fixed with 3% paraformaldehyde, washed thrice to remove excess paraformaldehyde and permeabilized by adding ice-cold methanol for 5 min at −20°C. Finally, cells were washed carefully with 1X PBS, mounted with fluoroshield (Sigma-Aldrich, USA) containing 4,6-diamidino-2-phenylindole (DAPI) and were viewed under Leica confocal microscope.

### Quantification of LTR activity in presence of transiently expressed hZNF-134

HEK293T cells were seeded one day prior to transfection. HEK293T cells were transfected with various plasmids as per the experimental requirement. Cells were washed with PBS to remove FBS and kept in media without FBS prior to transfection. DNA-lipid complex was prepared by mixing plasmids (pEGFP-C3 or ZNF-134-GFP-C3 or pcTAT), Lipofectamine LTX (Invitrogen) reagent and plus reagent (Invitrogen) in proper ratio according to manufacturer's protocol. The DNA-lipofectamine complex was kept at RT for 30 min followed by addition to the cells. For luciferase based assay, pLTR-luc (30 ng/well) and pRL-TK (5 ng/well) were co-transfected with pEGFP-C3 or ZNF-134-GFP-C3 (100 ng/well) or pcTAT (20 ng/well) separately or together. 48 hours post transfection, cells were harvested and analyzed for luciferase activity using Dual Glow Luciferase assay Kit as per the manufacturer's protocol (Promega, USA). Transfection efficiencies were normalized using pRL-TK. Firefly and Renilla luminescence readings were measured by single tube luminometer (Turner Biosystems, USA). The plots are denoted as fold increase in the luciferase activity as compared to control.

For quantification of the LTR driven viral transcripts, second round of transfection was performed with pNL4-3 vector (40 ng/well) after 24 hours of transfection of pEGFP-C3 or ZNF-134-GFP-C3 vector. The transfection efficiencies of pNL4-3 were normalized with pRL-TK. 48 hours post transfection, the cells were harvested and RNA was isolated for quantification of the viral transcripts using the TAR-FP and 9 kb-FP primers (Table S1) by qRT-PCR according to previously described protocol [59]. p24 equivalent measure of viral load was performed from the culture supernatant using Capture p24 ELISA kit (Advanced BioScience Laboratories Inc, USA).

### siRNA knockdown of hZNF-134

The DNA sequences used to design siRNA are 5′CCACAAATACACCCTCATT3′ for hZNF-134 and 5′CAGTCGCGTTTGCGACTGG 3′ for scrambled siRNA. The HEK293T cells were seeded and rested for 24 hours before hZNF-134 specific siRNA (50 picomoles) or scrambled siRNA (50 picomoles) were transfected using Lipofectamine RNAiMax reagent (Invitrogen, USA). In brief, cells were seeded at 60–80% confluency a day before transfection. Cells were washed with PBS and fresh media was added to the cells without FBS and antibiotics. Different amount of siRNA was diluted in serum free media and siRNA was added to diluted lipofectamine RNAiMax reagent in 1∶1 ratio. Mixture was incubated for 5–15 min and added to the cells slowly. The cells were kept for 48 hours before scoring for knockdown or cell death. Knockdown of *hznf-134* gene was measured after 48 hours of siRNA transfection by qRT-PCR and normalized with β-actin transcript levels. In similar experiment, after 24 hours of siRNA treatment, HEK293T cells were transfected with proviral DNA pNL4-3 (40 ng/well). 48 hours post transfection of pNL4-3, the cells were harvested to isolate RNA by Trizol reagent (Invitrogen, USA) and RNA levels were measured by qRT-PCR using RT ZNF FP and RT ZNF RP (Table S1). The transcript levels were normalized to the β-actin levels. *gapdh* was used as an additional control in siRNA experiments. qRT-PCR of *gapdh* transcripts was performed using the reported primers [61] and the levels of *gapdh* were normalized using β-actin mRNA levels. The culture supernatants were used to measure the viral load by p24 capture ELISA.

### Quantitative Real Time PCR (qRT-PCR)

RNA was isolated using Trizol reagent followed by cDNA preparation using superscript III (Invitrogen, USA) and oligo dT (Fermentas, Germany). Quantitative Real Time PCR (qRT-PCR) was performed using SYBR Premix Ex Taq mix (Takara Bio Inc, Japan) in Realplex2 Mastercycler (Eppendorf, Germany). The average threshold CT values were determined and normalized to β-actin mRNA levels which were used as an internal control. The fold difference was calculated using the formula 2^−(ΔΔCT)^.

### Statistical analyses and plots

All the experiments were repeated at the least three times. The data were plotted using Sigma plot software version 11.0.0.77 (USA). The error bars represent the standard deviation from the mean of at least three independent experiments. Statistical analyses of the experimental data were performed by Student's t-tests using Sigma Plot. p<0.05 was considered as significant.

## Supporting Information

Figure S1 **Biotinylated LTR immobilized to streptavidin agarose beads was used as bait to capture DNA binding proteins from Astrocytoma 1321N1 cell lysates.** (A) Schematic representation of HIV-1 LTR. The arrows indicate the position of Forward primer LTR-FP and Reverse primer LTR-RP-biotin, used for LTR amplification. (B) Agarose gel electrophoresis showing amplified LTR band corresponding to 634 bp. M denotes 100 bp ladder. (C) 1∶10 and 1∶100 dilutions of astrocyte cell lysates were used for pull-downs with biotinylated LTR. As a control, cell lysates were added to the beads without bound DNA. Samples were fractionated on 10% SDS-PAGE followed by silver staining of the gels. * indicates lanes which did not resolve properly; M denotes marker. Experiment was repeated more than three times. Representative gel is shown. (D) 1∶100 dilution of 1321N1 cell lysates (10 ug/ml) used for pull-down assays with biotinylated LTR captured limited number of proteins. Two independent pull-downs are shown here. ** indicates empty lane; M denotes marker. The arrows indicate the protein bands that were excised for MALDI analyses and later identified as hZNF-134.
(TIF)

Figure S2 **Mascot results.** (A) Tabulation of partial list of proteins identified through Mascot search from pull-down assays using biotinylated LTR as bait. (B) Mascot score Histogram showing significant score for Zinc finger protein-134.
(TIF)

Figure S3 **Schematic representation of domain organization of hZNF-134 protein.** The 11 C2H2 Zinc finger domains are highlighted and their positions are shown. The second C2H2 Zinc finger domain is degenerated and is shown as a pentagon.
(TIF)

Figure S4 **hZNF-134 transcript levels in different cell lines.** RT-PCR was performed on RNA isolated from 1321N1, GO-G-CCM, SUP-T1 and THP-1 cell lines using hZNF-134 and β-actin primers. The experiments were performed three times and representative gel is shown.
(TIF)

Figure S5 **MTT assay for hZNF-134 and scrambled siRNA treatment on HEK293T cells.** HEK293T cells were transfected either with 50 and 70 picomoles of hZNF-134 specific siRNA or 70 picomoles of scrambled siRNA (2 different sets) for 2 days. After 48 hours, % survival was measured by MTT assay where OD of scrambled siRNA was taken as 100% and others were converted accordingly.The values are average of three independent experiments and p value <0.05 were taken as significant and denoted as *.
(TIF)

Figure S6 **Transcripts levels of** ***gapdh*** **quantified by qRT-PCR under the treatment of the scrambled siRNA or hZNF-134 siRNA.** The change in transcript levels of *gapdh* normalized to the transcript levels of β-actin were found to be insignificant after treatment with either scrambled siRNA or hZNF-134 siRNA. All experiments were done more than three times and error bars represents mean ± SD. Student's t-test was performed and *p<0.05 was considered significant.
(TIF)

Figure S7 **hZNF-134 transcript levels in astrocytes and glial cell lines upon HIV infection.** qRT-PCR analysis shows increase in the transcript levels of hZNF-134 upon HIV-1 infection in astrocytes and glial cells. The transcript levels were normalized with β-actin transcripts.
(TIF)

Figure S8 **Cloning of hZNF-134-GFP-C3.** hZNF-134 was amplified from cDNA of astrocytoma 1321N1 cells and cloned into XhoI and BamHI sites of the pEGFP-C3 vector to generate ZNF-134-GFP-C3 construct. 1.5% agarose gel showing products after double digestion of ZNF-134-GFP-C3 construct. pEGFP-C3 linearized vector and hZNF-134 insert bands can be seen. M denotes 1 kb marker.
(TIF)

Table S1 **List of primers used in the study.** **Additional References.** 1. Chevallet M, Luche S, Rabilloud T: **Silver staining of proteins in polyacrylamide gels.** *Nat Protoc* 2006, **1:**1852–1858. 2. Garapati UK, Suryanarayana T: **Isolation of two strong poly (U) binding proteins from moderate halophile Halomonas eurihalina and their identification as cold shock proteins.** *PLoS One* 2012, **7:**e34409.s.
(DOCX)

File S1 (DOCX)
