# Supplementary figures and images for: Mycobacterial and HIV Infections Up-Regulated Human Zinc Finger Protein 134, a Novel Positive Regulator of HIV-1 LTR Activity and Viral Propagation
Source: PLoS One. 2014 Aug 21;9(8):e104908. doi: 10.1371/journal.pone.0104908 (PMC4140746; doi:10.1371/journal.pone.0104908)

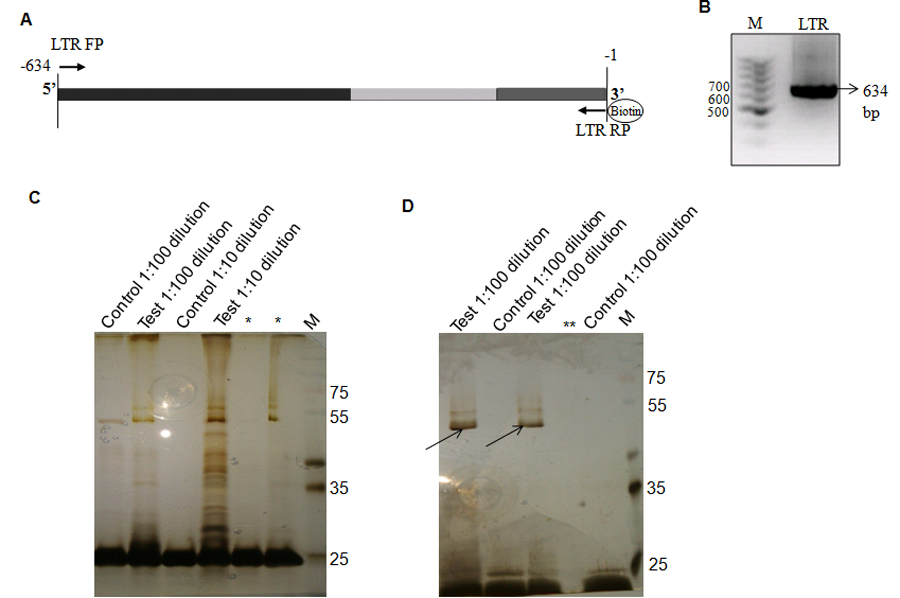

Supplement: Figure S1 — Biotinylated LTR immobilized to streptavidin agarose beads was used as bait to capture DNA binding proteins from Astrocytoma 1321N1 cell lysates. (A) Schematic representation of HIV-1 LTR. The arrows indicate the position of Forward primer LTR-FP and Reverse primer LTR-RP-biotin, used for LTR amplification. (B) Agarose gel electrophoresis showing amplified LTR band corresponding to 634 bp. M denotes 100 bp ladder. (C) 1∶10 and 1∶100 dilutions of astrocyte cell lysates were used for pull-downs with biotinylated LTR. As a control, cell lysates were added to the beads without bound DNA. Samples were fractionated on 10% SDS-PAGE followed by silver staining of the gels. * indicates lanes which did not resolve properly; M denotes marker. Experiment was repeated more than three times. Representative gel is shown. (D) 1∶100 dilution of 1321N1 cell lysates (10 ug/ml) used for pull-down assays with biotinylated LTR captured limited number of proteins. Two independent pull-downs are shown here. ** indicates empty lane; M denotes marker. The arrows indicate the protein bands that were excised for MALDI analyses and later identified as hZNF-134. (TIF) [file pone.0104908.s001.tif]

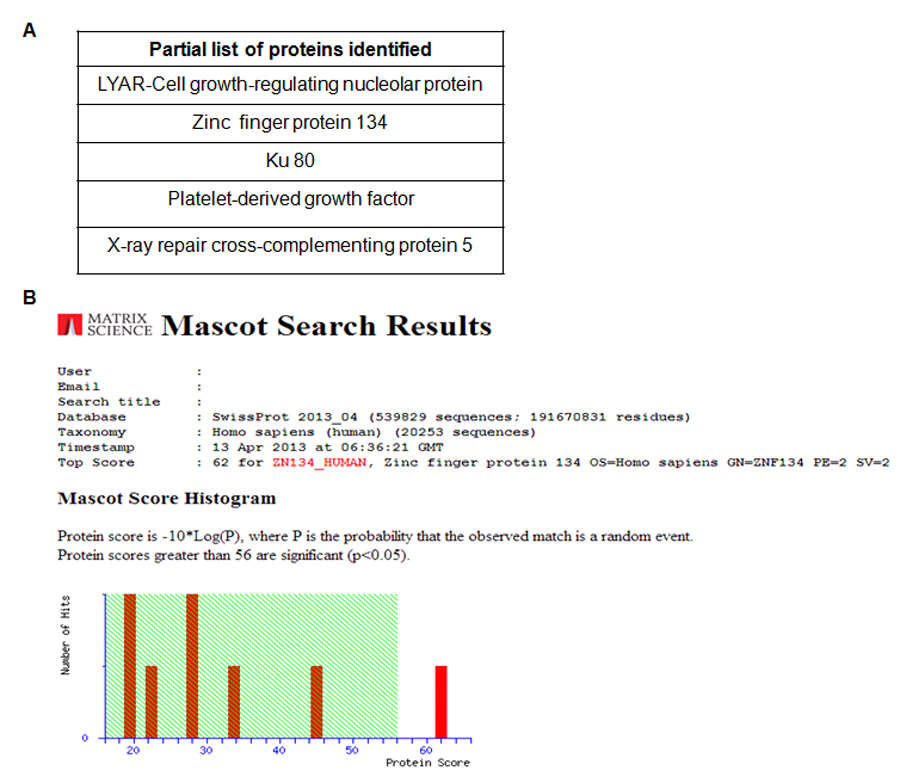

Supplement: Figure S2 — Mascot results. (A) Tabulation of partial list of proteins identified through Mascot search from pull-down assays using biotinylated LTR as bait. (B) Mascot score Histogram showing significant score for Zinc finger protein-134. (TIF) [file pone.0104908.s002.tif]

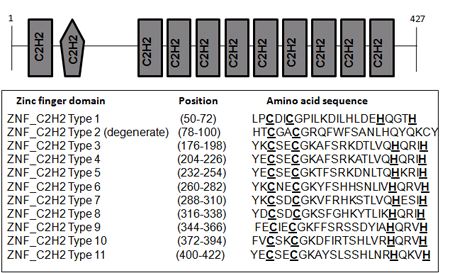

Supplement: Figure S3 — Schematic representation of domain organization of hZNF-134 protein. The 11 C2H2 Zinc finger domains are highlighted and their positions are shown. The second C2H2 Zinc finger domain is degenerated and is shown as a pentagon. (TIF) [file pone.0104908.s003.tif]

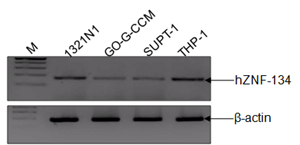

Supplement: Figure S4 — hZNF-134 transcript levels in different cell lines. RT-PCR was performed on RNA isolated from 1321N1, GO-G-CCM, SUP-T1 and THP-1 cell lines using hZNF-134 and β-actin primers. The experiments were performed three times and representative gel is shown. (TIF) [file pone.0104908.s004.tif]

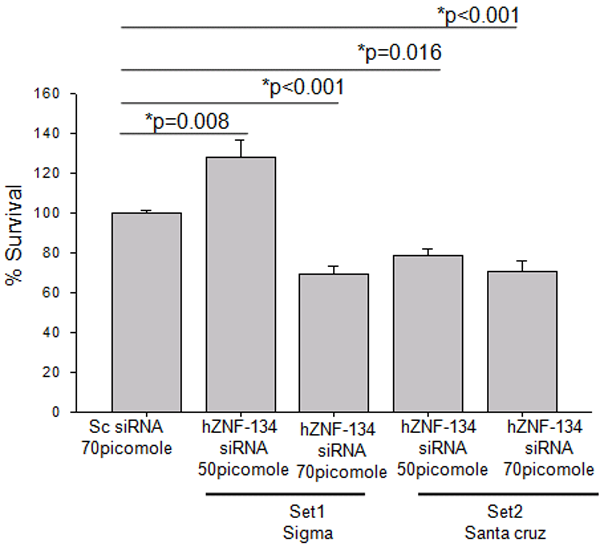

Supplement: Figure S5 — MTT assay for hZNF-134 and scrambled siRNA treatment on HEK293T cells. HEK293T cells were transfected either with 50 and 70 picomoles of hZNF-134 specific siRNA or 70 picomoles of scrambled siRNA (2 different sets) for 2 days. After 48 hours, % survival was measured by MTT assay where OD of scrambled siRNA was taken as 100% and others were converted accordingly.The values are average of three independent experiments and p value <0.05 were taken as significant and denoted as *. (TIF) [file pone.0104908.s005.tif]

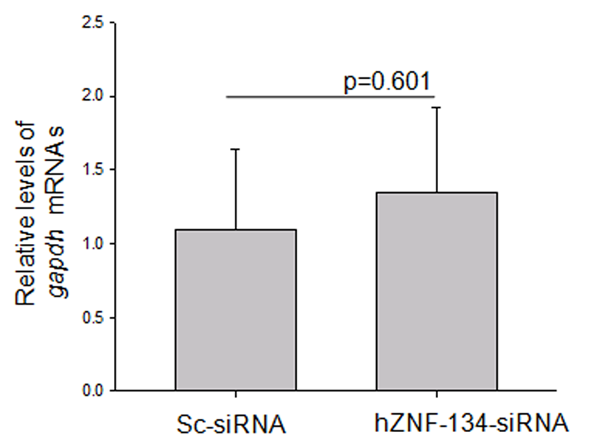

Supplement: Figure S6 — Transcripts levels of gapdh quantified by qRT-PCR under the treatment of the scrambled siRNA or hZNF-134 siRNA. The change in transcript levels of gapdh normalized to the transcript levels of β-actin were found to be insignificant after treatment with either scrambled siRNA or hZNF-134 siRNA. All experiments were done more than three times and error bars represents mean ± SD. Student's t-test was performed and *p<0.05 was considered significant. (TIF) [file pone.0104908.s006.tif]

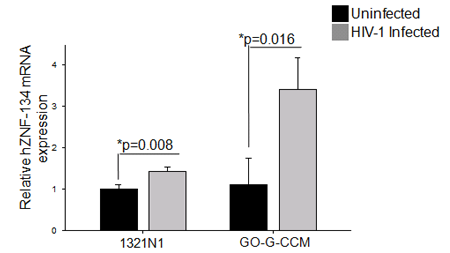

Supplement: Figure S7 — hZNF-134 transcript levels in astrocytes and glial cell lines upon HIV infection. qRT-PCR analysis shows increase in the transcript levels of hZNF-134 upon HIV-1 infection in astrocytes and glial cells. The transcript levels were normalized with β-actin transcripts. (TIF) [file pone.0104908.s007.tif]

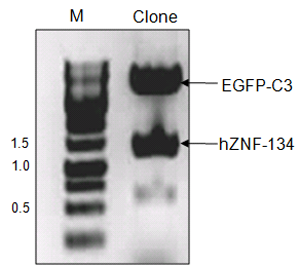

Supplement: Figure S8 — Cloning of hZNF-134-GFP-C3. hZNF-134 was amplified from cDNA of astrocytoma 1321N1 cells and cloned into XhoI and BamHI sites of the pEGFP-C3 vector to generate ZNF-134-GFP-C3 construct. 1.5% agarose gel showing products after double digestion of ZNF-134-GFP-C3 construct. pEGFP-C3 linearized vector and hZNF-134 insert bands can be seen. M denotes 1 kb marker. (TIF) [file pone.0104908.s008.tif]
