# Supplementary material for: Mycobacterial and HIV Infections Up-Regulated Human Zinc Finger Protein 134, a Novel Positive Regulator of HIV-1 LTR Activity and Viral Propagation
Source: PLoS One. 2014 Aug 21;9(8):e104908. doi: 10.1371/journal.pone.0104908 (PMC4140746; doi:10.1371/journal.pone.0104908)
Supplement: Table S1 — List of primers used in the study. Additional References. 1. Chevallet M, Luche S, Rabilloud T: Silver staining of proteins in polyacrylamide gels. Nat Protoc 2006, 1:1852–1858. 2. Garapati UK, Suryanarayana T: Isolation of two strong poly (U) binding proteins from moderate halophile Halomonas eurihalina and their identification as cold shock proteins. PLoS One 2012, 7:e34409.s. (DOCX) [file pone.0104908.s009.docx]

| **Pairwise number** | **PRIMER NAME** | **PRIMER SEQUENCE** |
| --- | --- | --- |
| 1st | LTR FP | 5’ TGGAAGGGCTAATTTGGTCCCAAAAAAG 3’ |
|  | LTR RP Biotin | 5’ TTAGTCAGTGTGGAAAATCTCTAGCA 3’ |
| 2nd | ZNF GFP FP | 5’ GAGCTCGAGATGACTCTAGTCACAGCAGGAGGGGCT 3’ |
|  | ZNF GFP RP | 5’ GTAGGATCCCTAAAGCCTGCCTGCAGTGTGAACTTTCTG 3’ |
| 3rd | RT ZNF FP | 5’ TGACTCTAGTCACAGCAGGAGG 3’ |
|  | RT ZNF RP | 5’ ACATATGTCACAAGGGAGAGCCGTCTG 3’ |
| 4th | RT ACTIN FP | 5’ AGCCTCGCCTTTGCCGA 3’ |
|  | RT ACTIN RP | 5’ CTGGTGCCTGGGGCG 3’ |
| 5th | GAPDH FP | 5′ GTCCCCTCGAGGAGTTGTGT 3′ |
|  | GAPDH RP | 5′ ATCTTCCATCATCTGAGGGC 3′ |
| 6th | TAR-FP | 5’ CTGAGCCTGGGAGCTCTCTGGC 3’ |
|  | 9Kb-RP | 5’ TGCGAATCGTTCTAGCTCCCTGCTTGCCCATACTATATGTTT 3’ |

**Supporting table S1: List of primers used in the study**
