# Supplementary material for: Mycobacterial and HIV Infections Up-Regulated Human Zinc Finger Protein 134, a Novel Positive Regulator of HIV-1 LTR Activity and Viral Propagation
Source: PLoS One. 2014 Aug 21;9(8):e104908. doi: 10.1371/journal.pone.0104908 (PMC4140746; doi:10.1371/journal.pone.0104908)
Supplement: File S1 — (DOCX) [file pone.0104908.s010.docx]

**Supporting data**

**Methods and Materials:**

**Pull-down from astrocytoma 1321N1 cell lysates using biotinylated HIV-1 LTR.** A 634bp long HIV-1 LTR was amplified by Jumpstart Accutaq polymerase (Sigma-Aldrich, USA) from the template plasmid, pNL4-3 using the forward primer LTR-FP and the biotinylated reverse primer, LTR-RP-Biotin (Table S1). The amplified products were purified by DNA purification columns (Qiagen PCR Purification Kit, Germany) to eliminate excess of free primers. 60µl of *Streptavidin* Agarose beads (Invitrogen, USA) were equilibrated by washing with 1X TE buffer (10mM Tris-HCl, 1mM EDTA pH 8). 20pmol of biotinylated LTR was added to the beads in 1X TE buffer and kept for 3 hours at 25°C on rocker. After 3 hours, unbound biotinylated LTR were removed by 3 stringent washes with 1X SSC buffer (saline sodium citrate buffer, pH 7).

As a large number of cellular proteins are known to bind LTR, we diluted the whole cell lysates into 1:10 and 1:100 dilutions with PBS. The lysates were then divided into two equal aliquots. 60µl Streptavidin Agarose beads were added to one of the aliquots as a bead control, while 60µl of biotinylated LTR immobilized on Streptavidin Agarose beads were added to the second aliquot. The binding was performed overnight at 4°C on rocker. After overnight incubation, the beads were washed 5 times with 1X SSC buffer. 100µl of SDS loading dye was added to the beads and samples were boiled for 10 min at 95°C followed by fractionation of 20µl of sample on 10% SDS PAGE. The gels were silver stained by using previously described protocol [1]. Pull-down with 1:10 lysates showed several bands (Figure S1C, lane 4), while pull-downs with 1:100 lysates showed few protein bands (Figure S1C lanes 2, S1D lane 1 and 3). The protein bands were excised from different gels and subjected to MALDI analyses as described earlier [2] (BrukerDaltonics, Bremen, Germany) at the proteomics facility of University of Hyderabad to identify the proteins. Some of the proteins that were repeatedly identified in our conditions are enlisted in Figure S2A, amongst which was human Zinc Finger Protein 134 (hZNF-134). Protein identification was carried out by database searches (PMF and MS/MS) using MASCOT program (http://www.matrixscience.com) employing Biotools software (BrukerDaltonics). The MASCOT score with the histogram for hZNF-134 is shown in Figure S2B.

# MTT assay for cell viability

# Cell viability was checked after 48 hours of siRNA treatment by 3-(4,5-Dimethylthiazol-2-yl)-2,5-diphenyltetrazolium bromide (MTT) assay. HEK293T cells were transfected either with 50 and 70picomoles of hZNF-134 specific siRNA or 70picomoles of scrambled siRNA. After 48 hours of siRNA treatment, the tetrazolium dye, MTT (5mg/ml) was added to each well and kept for 4 hours incubation at 37°C. The purple formazan product was dissolved in DMSO and OD was read at 570nm. The OD values are converted into % survival by considering OD of the scrambled siRNA as 100% and plotted by using Sigma plot (software version 11.0.0.77, USA). Three independent experiments were performed.

**Figure legends**

**Figure S1**

**Biotinylated LTR immobilized to streptavidin agarose beads was used as bait to capture DNA binding proteins from Astrocytoma 1321N1 cell lysates.** (A) Schematic representation of HIV-1 LTR. The arrows indicate the position of Forward primer LTR-FP and Reverse primer LTR-RP-biotin, used for LTR amplification. (B) Agarose gel electrophoresis showing amplified LTR band corresponding to 634bp. M denotes 100bp ladder. (C) 1:10 and 1:100 dilutions of astrocyte cell lysates were used for pull-downs with biotinylated LTR. As a control, cell lysates were added to the beads without bound DNA. Samples were fractionated on 10% SDS-PAGE followed by silver staining of the gels. * indicates lanes which did not resolve properly; M denotes marker. Experiment was repeated more than three times. Representative gel is shown. (D) 1:100 dilution of 1321N1 cell lysates (10ug/ml) used for pull-down assays with biotinylated LTR captured limited number of proteins. Two independent pull-downs are shown here. ** indicates empty lane; M denotes marker. The arrows indicate the protein bands that were excised for MALDI analyses and later identified as hZNF-134.

**FigureS2**

**Mascot results.** (A) Tabulation of partial list of proteins identified through Mascot search from pull-down assays using biotinylated LTR as bait. (B) Mascot score Histogram showing significant score for Zinc finger protein-134.

**Figure S3**

**Schematic representation of domain organization of hZNF-134 protein.** The 11 C2H2 Zinc finger domains are highlighted and their positions are shown. The second C2H2 Zinc finger domain is degenerated and is shown as a pentagon

**Figure S4**

**hZNF-134 transcript levels in different cell lines.** RT-PCR was performed on RNA isolated from 1321N1, GO-G-CCM, SUP-T1 and THP-1 cell lines using hZNF-134 and β-actin primers. The experiments were performed three times and representative gel is shown.

**Figure S5**

**MTT assay for hZNF-134 and scrambled siRNA treatment on HEK293T cells.**HEK293T cells were transfected either with 50 and 70 picomoles of hZNF-134 specific siRNA or 70 picomoles of scrambled siRNA (2 different sets) for 2 days. After 48 hours, % survival was measured by MTT assay where OD of scrambled siRNA was taken as 100% and others were converted accordingly.The values are average of three independent experiments and p value <0.05 were taken as significant and denoted as *.

**FigureS6**

**Transcripts levels of *gapdh* quantified by qRT-PCR under the treatment of the scrambled siRNA or hZNF-134 siRNA.** The change in transcript levels of *gapdh* normalized to the transcript levels of β-actin were found to be insignificant after treatment with either scrambled siRNA or hZNF-134 siRNA. All experiments were done more than three times and error bars represents mean ± SD. Student’s t-test was performed and *p<0.05 was considered significant.

**Figure S7s**

**hZNF-134 transcript levels in astrocytes and glial cell lines upon HIV infection.** qRT-PCR analysis shows increase in the transcript levels of hZNF-134 upon HIV-1 infection in astrocytes and glial cells. The transcript levels were normalized with β-actin transcripts.

**Figure S8**

**Cloning of hZNF-134-GFP-C3.** hZNF-134 was amplified from cDNA of astrocytoma 1321N1 cells and cloned into XhoI and BamHI sites of the pEGFP-C3 vector to generate ZNF-134-GFP-C3 construct. 1.5% agarose gel showing products after double digestion of ZNF-134-GFP-C3 construct. pEGFP-C3 linearized vector and hZNF-134 insert bands can be seen. M denotes 1kb marker.

**Table S1**

List of primers used in the study

**Additional References**

1. Chevallet M, Luche S, Rabilloud T: **Silver staining of proteins in polyacrylamide gels.***Nat Protoc* 2006, **1:**1852-1858.

2. Garapati UK, Suryanarayana T: **Isolation of two strong poly (U) binding proteins from moderate halophile Halomonas eurihalina and their identification as cold shock proteins.***PLoS One* 2012, **7:**e34409.s
